# Experiences of Young People and Their Carers with a Rural Mobile Mental Health Support Service: A Qualitative Study

**DOI:** 10.3390/ijerph20031774

**Published:** 2023-01-18

**Authors:** Julaine Allan, Anna Thompson

**Affiliations:** 1 Rural Health Research Institute, Charles Sturt University, Orange, NSW 2800, Australia; 2 School of Rural Health, Faculty of Medicine and Health, University of Sydney, Orange, NSW 2800, Australia

**Keywords:** rural, mental health, adolescents, access, service user experience, interventions

## Abstract

Mental health difficulties during teenage years are common. They are also a risk factor for later mental and physical health problems. Rural young people are at a greater risk for mental health difficulties and have less access to services than their urban counterparts. The purpose of this study was to explore the experiences of young people and their carers with mental health support provided by a rural mobile service, and to identify access enablers from the perspective of the service users. A qualitative descriptive approach was used to analyse twelve interviews with current service users and eight interviews with family members of young people who had accessed the service. Three main themes were identified: (a) access and flexibility, (b) the qualities and strategies of the clinicians, and (c) experiences of change. The mobile service was perceived to be effective in producing a positive change in mental health, relationships, and the attainment of life goals. Key enablers to access included the flexibility of the mobile service, the variety of service delivery modes and therapeutic methods offered, the ease of access facilitated by the location in schools, and the autonomy of young people in how they chose to utilise the service. This study provides information about what is important to rural young people and their families in the provision of mental health services. The findings have implications for changing the way services are organized and operated. Healthcare policies and services could support a user-led model design that incorporates access and use-enablers and removes barriers to rural mental health support.

## 1. Introduction

The teenage years are an important time of social, emotional, and physical development. One in six people in the world are young people, and it is estimated that one in seven of them experiences a mental health condition, most of which are untreated [1]. Mental health problems experienced during this time are a key risk factor for both mental and physical health problems in adulthood [2]. The importance of addressing the mental health conditions of young people is recognised in global strategies for attaining the Sustainable Development Goals (SDGs). For example, Goal Three states that it is important to “ensure healthy lives and promote wellbeing for all *at all ages* including coverage of essential health services such as mental health care” [3].

In Australia, young people are a priority for the provision of mental health services, including suicide prevention [4]. Australian young people living outside capital cities are at a higher risk of mental distress than those living in capital cities due to the unique circumstances of rural areas. For example, disasters such as floods, drought, and bushfires compound the effects of high unemployment rates, low incomes, and a lack of resources, particularly in health and education [5]. The most recent national survey published in 2015 [4] identified that 16.4% of rural young people aged four to seventeen years reported mental health conditions. The most common were ADHD, anxiety, and depression. Further, the study found that the risk of suicide increases with remoteness. This is consistent with evidence that the lower the mental health treatment capacity in an area, the greater the risk of suicide in young people. The mental health challenges of Australian young people in rural areas are exacerbated by shortages in the mental health workforce and the difficulty in accessing appropriate services [6]. The rural mental health workforce shortage exists across all medical and allied health staff [7].

### 1.1. Service Models and Innovations

Solutions to supply problems in the rural health workforce are sought globally [8]. The World Health Organization has recommendations for policy initiatives to address workforce maldistribution in high income countries, including education strategies, regulatory change, financial incentives, and personal and professional supports [5]. At a local level, rural health care organisations manipulate budgets, staff, and infrastructure to provide as many services as they can afford, balancing between community demand, assessed need, and workforce supply [9,10]. In Australia, many support and allied health services in smaller and more remote rural towns are provided by clinicians visiting on a fortnightly to quarterly basis [11]. This is a strategy to cover large areas with low referral rates and few specialist staff.

Australian governments have devolved the responsibility of supplying many health and community services to the non-government sector, using competitive tendering processes to select and fund those organisations that offer innovation, value for money, and a stated capability to resource specific locations. In mental health services, a stepped approach is used, in which prevention, primary care, early intervention, and services for specific groups such as young people are tendered out, and the public health system provides tertiary-level care in hospitals and community support for those with a serious mental illness [12]. Virtual support services providing information, applications with meditation exercises, for example, and real-time access to clinicians have been growing as one way of addressing access barriers and making mental health support more accessible [13,14]. Reports of user satisfaction and the outcomes of virtual care vary [15,16]. However, given the move to virtual care due to the COVID-19 pandemic, it is likely that virtual services will become more common as people grow accustomed to using them and clinicians become more experienced in providing them. The selection of an appropriate physical location for an in-person intervention is also a focus. Locating mental health services in schools as a strategy to increase access to the services is common in the U.K. and U.S.A. [17,18,19]. In Australia, health care services within schools—as opposed to a school counsellor who makes referrals—is a recent initiative, and its impact is largely unevaluated [20,21].

### 1.2. Voices of Young People Regarding Mental Health Services

Data regarding the mental health service delivery experience of rural young people in Australia is limited [6]. While clinician availability is a critical supply challenge in rural Australia, it is not the only reason that adolescents do not access mental health support [22]. A recent systematic review searched for first-hand accounts of the experiences of mental health services from children and young people (0–17 years of age);it only included eleven papers published in English worldwide since 2000 [23]. None of the included reports included a rural focus or considered rural-specific conditions in the study. However, key themes identified across studies included the importance of relationships, accessibility, and user involvement as enablers of mental health support [23]. Other studies inclusive of wider age ranges have identified related barriers. Concerns about being judged by the clinician and the community, extended wait times and complicated assessment processes, lack of knowledge about privacy and confidentiality or its limits (informing family of details, for example), and a lack of connection with the clinician have all been identified as barriers to the access of mental health support by young people [22,24,25,26]. Ideally, the concerns and preferences of young people will drive the design and operation of support services to address these barriers [27].

Given the additional challenges faced by rural young people in finding support for their mental health concerns, the aim of this study was to explore the experiences of young people and their carers in relation to mental health support provided by a rural, mobile team of clinicians. This study also aimed to identify barriers and enablers of access from the perspectives of the service users.

## 2. Method

### 2.1. Setting and Design

Western New South Wales (NSW) covers an area of 433,379 square kilometres and has a widely dispersed population of around 317,000 people living in regional centres, towns, villages, and on farming properties [28]. Approximately thirteen per cent of the population are First Nations Australians, compared to three per cent in all of NSW [29]. Health services are centralised in the larger regional towns, with multi-purpose services (including aged care and emergency departments) in the smaller towns. The Western NSW Primary Health Network 2019–2022 Health Needs Assessment [30] identified mental health services as the most important need for the region. In response, a non-government organisation was funded by the Commonwealth Government to provide an outreach service to young people across a large part of western NSW (in twelve local government areas [LGAs]).

The service objectives were to provide easily accessible mental health services in schools and other community facilities for young people aged 12 to 25 years, linking with other agencies and community networks in each town and being culturally responsive to First Nations Australians seeking support. The service was commissioned to work across all the stages of stepped care and was intended to be easily accessible, developmentally appropriate, and responsive to the mental health conditions or concerns of those using it. Referrals to the service could be made by anyone, including young people, via phone, email, or in-person. There were no specific criteria or diagnoses required to attend, aside from age and having residence in the area. The area was divided into regions with three or four sites in each region. Clinicians employed by the service attended each of the sites in their designated region weekly or fortnightly for one or two days, depending on demand. Young people could attend the service as many times as they wished. Young people under the age of sixteen were required to have parental or carer consent to attend the service.

To explore the experiences of young people and their carers with the mobile mental health support service, a qualitative descriptive approach [31] was used. Qualitative description aims to represent the experience of people with integrity [32]. The method can provide the reader with an understanding of the interpretations of research participants with respect to how and why an event took place, rather than providing a researcher-interpreted meaning [33]. In this study, the accounts from participants explained how they experienced the service and whether it met their needs. The established service model provided a framework for the data collection and analysis in that the interview questions and analysis asked about access, appropriateness, responsiveness, and the capability to deal with the concerns of the young person.

Ethical approval for the study was obtained from the Aboriginal Health and Medical Research Council of NSW–Human Ethics Research Committee (1830/21).

### 2.2. Recruitment and Sample Characteristics

Purposive sampling was used to recruit participants, who were (a) current clients of the service and had consented to be contacted for feedback or (b) family members/carers of current clients who had given permission for their family member/carer to be contacted. Invitations to participate were emailed by service clinicians or given to clients at their appointment during September 2021. Interested people contacted the research team directly by phone or email. Participation was voluntary and anonymous. Participants were offered an AUD 30 gift voucher. The amount was considered to not be an inducement to participate, but did provide some compensation for their time.

Twelve interviews were conducted with young people who accessed the service. Eight interviews were conducted with the family members or carers of young people who accessed the service. Five of the young people interviewed also had a family member participate. The young people interviewed were aged between 13 and 17 years. One was male, the remainder were female. One young person identified as a First Nations Australian. The young-people participants came from seven of the twelve LGAs visited by the service. They had been engaged with the service for an average of 6.5 months (in a range of one to fifteen months). The majority (nine) had been referred by a staff member at school (school counsellor, wellbeing teacher, or other teacher), two were referred by family, and one had self-referred. All had received individual services; no young people reported participating in any groups as part of their contact with the service. All eight of the family members interviewed were mothers of young people currently accessing the service. Two of the mothers had two children who had accessed the service. The families had been involved with the service for an average of seven months (in a range of one to eighteen months). The family members came from five of the twelve LGAs visited by the service. None of the family members identified themselves or their child as a First Nations Australian.

The sample of young people was consistent with the total service-user population in relation to age, with 94% of clients being 13–18 years of age (2020/21 Annual Report). However, it was skewed toward female participants, with only one male out of 12 participants (8%), whereas 23% of service users are male. With only one First Nations young person, the sample also underrepresented First Nations service users, who make up 49% of the total client group.

### 2.3. Data Collection

The semi-structured interview guide was developed by both authors based on the objectives of the service. It included open-ended questions such as, “Tell me how you got in touch with [clinician]?” and, “What is the most important thing a counsellor/service needs to know about working with someone your age facing problems like yours?” In addition, prompts were utilised to encourage participants to reflect and expand on their experiences. Family members were asked questions about the family experience with the service, whether anything changed for the young person or family because of the contact, and whether there was anything they would change about the service. Due to COVID-19 restrictions on contact and travel, interviews were conducted by phone or online using Zoom, lasting between 15 and 50 min. Interviews were conducted by A.T., a clinical psychologist with extensive experience working in rural mental health and conducting mixed-methods research. With the consent of participants, interviews were audio-recorded, professionally transcribed, and de-identified.

### 2.4. Data Analysis

The data analysis was conducted by J.A. and A.T. J.A. has more than twenty years of experience in qualitative rural health research with vulnerable groups, particularly examining the impact of government and service-delivery policies and practices on service users. Debrief meetings, held after two or three interviews were conducted, were used to discuss and make notes on key themes or events described, as well as to ensure that there was consistency between the phone and online interviews. A.T. manually coded the transcribed data into themes using a deductive approach guided by the objectives of the service model. Specifically, the following questions were asked of the data: how was accessing the service described, how were the responsiveness and skills of the clinician described, what links were made with other local supports and community resources, and what were the perceptions of the participants of the benefits to and changes in their mental health? Finally, any problems experienced by study participants with the service were noted. Informational redundancy was used to determine when enough participants had been recruited, in that no new themes related to the study aim were identified in the analysis [34,35]. Further, because the study aim was narrow in that the access to and experience of a specific service in a defined area by a specific group of people was the phenomenon of interest, a small sample was appropriate [36].

The final themes were supported and confirmed through a review of the interview debriefing notes and discussions between J.A. and A.T. that focused on ensuring the lived experiences of the participants were reflected in the analysis, consistent with the qualitative description method and ensuring interpretive validity [33,37].

## 3. Results

**Figure 1:**
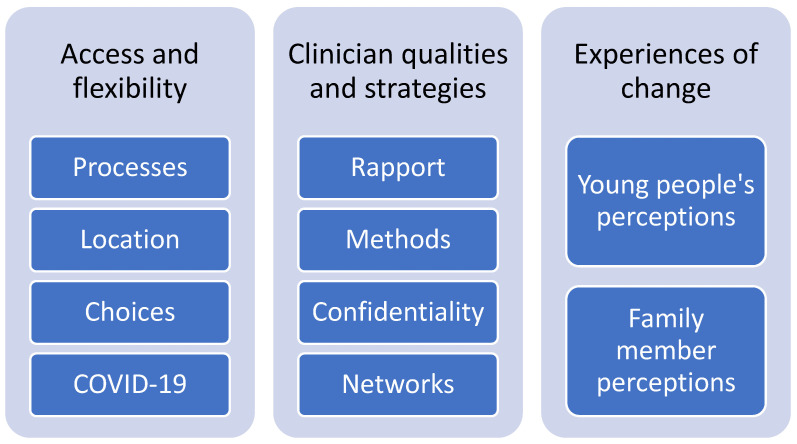
Summary of themes and sub-themes.

Three main themes were identified from the interviews: (1) access and flexibility; (2) clinician qualities and strategies; and (3) experiences of change. To protect the confidentiality of participants, they will be referred to as “young person” (YP) or “family member” (FM) and, when quoted, will be identified by their assigned number. Figure 1 summarizes the themes and sub-themes.

### 3.1. Access and Flexibility

Ease of access to and the flexibility of service provision were identified by all participants as critical elements of service operations. Multiple examples were provided of the ways that the needs and preferences of young people and their family members were accommodated. The COVID-19 pandemic forced some restrictions on service delivery during the 12-month data inclusion period, affecting access to the service. Statements about online service delivery were considered separately in the analysis.

### 3.2. Processes

The way the service operated was described as flexible and responsive to the needs of the young people. Referral pathways were described as simple and straightforward, without the need for specialist assessments or multiple steps. For example:

*It was a super easy process. I was meeting with [Youth Worker at school], and then just one day, she was like, “Oh, this is [clinician]. You can pick whether you want to see her, and she can help with strategies and stuff like that.” So for me, it was like a super easy process.*

(YP11)

Changing or missing appointments can often result in months of waiting when rural services are in short supply. However, examples were provided about how this was quickly addressed by the service:

*[YP] did forget the [appointment] one day and she sent [clinician] an email immediately once she realised, and [clinician] was like, “Oh, it’s okay. I’ve got a spot open this afternoon, would you like to meet?”, so it didn’t make her feel as bad, because [YP] is a worrier, she worries a lot.*

(FM07)

One family member compared the service to other services that were less flexible:

*She knows that she’s got someone to reach out to and talk to. … It’s built a lot of trust, I think for [YP] and she’s not feeling so isolated and alone waiting for her counselling appointment.…There’s no certain [number of] visits. There’s no “Okay I can only fit you in three times” or “You’ve got to wait two months before your next appointment”.*

(FM04)

Some young people did not want their parents to know they were seeking help. One identified that she could access the service independently because it was free:

*I like the fact it was free. Because the fact that we don’t have to pay for it makes me feel really good…. makes it feel a lot more accessible…, which I find really good. If it wasn’t [free] I would have to ask my parents, and I don’t want my parents to know about it.*

(YP06)

Several family members also noted that cost was a barrier in accessing healthcare and support, describing limited family finances and the high cost of accessing specialist healthcare. *This being a free service certainly makes it a lot more accessible than what otherwise could be.* (FM06).

### 3.3. Location

Most services were provided in schools. The *location* was easy to access for young people, particularly when they were inexperienced in help-seeking and did not know how to navigate the healthcare system:

*Just getting to like, find out about them. I found out through school, and I don’t know how else I’d find out about it.*

(YP02)

Family members identified additional benefits of the service location. Several participants stated that the location negated the need for the long-distance travel that is common in rural areas when accessing health care, taking time off work, and the costs associated with both the travel and services:

*We’re located 40 kms from our local town… My husband works full-time and I work part-time so being able to access those services has been tricky. And that’s what’s great about [service]-the fact that [clinician] goes to the school, so we don’t have to be concerned about how [YP] is going to get to their appointment and whether we have to take time off work or whatever.*

(FM8)

### 3.4. Choices

The service offered young people choices about when, where, and how they received support. For example, choosing the time of day; *Just easy to access … It’s pretty much whatever time suits me* (YP05) or the location: *It was at a cafe, but we started seeing each other more at school* (YP05). One young person described how the clinician would work around what was important to her at school: *If I had classes I wanted to go to because it was an in-school thing, she would work around it and stuff* (YP06). This did not mean that the service was available every day in every location, but the way the young people described the accessibility is notable: not in terms of lack of access or waiting for support, but rather as available and flexible. Young people described having a sense of control over how they accessed the service: *They gave me options of how much I got to see them* (YP02).

Family members also identified the perception of choice and responsiveness to each young person:

*I couldn’t believe that she met out of office hours. They did meet up at a cafe to talk. She provided support to get her to work, which was wonderful being out of town. It wasn’t a big journey, but just having that flexibility again, building that trust, building that [sense of] ‘I’ve got someone who is going to be there at all different times’.*

(FM01)

### 3.5. COVID-19

COVID restrictions had had little or no impact on the support young people perceived that they received from the service. For example: *It [COVID] wasn’t a big deal. It didn’t change the support I got from her [clinician]* (YP12). While the mode of contact changed for almost all young people from face-to-face to phone or Zoom when regional NSW entered a lockdown period in August 2021, the young people continued to experience the clinicians as available and supportive, and had the same frequency of contact.

*The actual conversations we have [on Zoom] are the normal conversations we usually have. And she can show me, like if she’s ever doing the drawings or like writing up stuff, she shows me what she writes. It doesn’t change anything that way at all.*

(YP06)

Preferences for on-line or face-to-face contact varied from person to person. Some young people preferred face-to-face contact, as in the pre-COVID period:

*I think in-person makes a bigger difference, and I like having it in person. You can read your body language more, you can read hers [clinician’s] more.*

(YP11)

However, others preferred on-line contact:

*I find it more easy to talk over Zoom but that’s just me. I don’t really like talking in person.*

(YP08)

The main problems noted with the on-line sessions were poor internet connections, which are common in rural Australia:

*Sometimes it’s annoying to do it over like over video call because my internet is absolutely horrid.*

(YP01)

A lack of privacy at home was also identified as a concern:

*I feel people are hearing my conversations [when I’m on a Zoom call at home]. I don’t want my family hearing them much.*

(YP10)

The parents interviewed also expressed that COVID restrictions over the 2020–2021 period had had a minimal impact on the support their young person received from the service. Some family members, noting their child’s preference for face-to-face contact, were concerned that the support would stop altogether. However, most were relieved to find that the support continued:

*I think that even though [YP] would like to meet [clinician] face-to-face sometimes, I feel like it’s still been good for her to meet over Zoom. It’s still been really helpful.*

(FM07)

Parents perceived that the accessibility of the clinicians—for example, in communication by text message—meant that there was a minimal impact of COVID on the support their young person was receiving:

*No, not at all because [clinician] has actually checked in once or twice a week with [YP]. I don’t feel that it has been impacted. And again, [YP] knows that [clinician] is just a text message away.*

(FM01)

One young person who did not want to engage in on-line or phone contact continued to have contact with the service:

*[YP] has had the option to do an over-the-phone with [clinician] since all this COVID stuffs happened, but she didn’t want to do the over-the-phone thing… she seems quite happy with waiting to go back to school… They were working on a drawing thing and [clinician] dropped that off at my work for me so I could take it home to [YP], and then she could just do stuff with it if she wanted to.*

(FM04)

### 3.6. Clinician Qualities and Strategies

A mental health support service must be supplied by appropriately skilled clinicians to be able to implement evidence-based interventions. A strong therapeutic relationship and effective methods for improving mental health are critical to good service provision. None of the study participants described their encounters with the service clinician in technical terms, such as referring to the use of Cognitive Behavioural Therapy. Rather, they described the building of rapport, the use of varied methods, and the facilitation of access to other services.

### 3.7. Rapport

The young people experienced the service clinicians as highly supportive, kind, and approachable individuals who listened:

*I feel like [clinician] is a very kind lady… and she’s very understanding and stuff, so I feel like that makes it a lot easier to be able to talk to her.* (YP04)

Rapport was enhanced because the young people perceived the clinicians as able to offer them plenty of time:

*They [clinician] are very friendly, you feel like you can approach them. They don’t rush you and they’re very patient, which is really good. Like, they give you time and they’re really understanding.*

(YP02)

The young people described multiple ways in which the service engaged with them that helped to build rapport and were appropriate for the age group. They described being able to have contact with their worker by phone, SMS, email, and videoconference (Zoom and Microsoft Teams), as well as face-to-face. *I have her [clinician] email. If I ever just feel really bad, she said just give her a little email of what’s happened* (YP10). Their experience of support was heightened by the ability to contact the clinician between sessions: *When I haven’t been feeling so good, she’s always been willing to call and stuff and talk and that* (YP03). Family members reported a similar experience. *[Clinician] being available to me too has been really good. Yeah, she was very adamant in saying that we could ring her and if we had a problem that was really urgent, she doesn’t mind if we text her either.* (FM02).

### 3.8. Methods

As well as having a rapport with the clinician, young people described the therapeutic methods used as part of the effectiveness of the service:

*I learned a lot of strategies, to help control my stress and my anxiety that I can use in everyday life, and it makes me less anxious. I get the worst anxiety when I’m in a group setting, and she’s helped me to know… the things that I can do to help me calm myself down, and not get as anxious around people.*

(YP11)

The young people appreciated the variety of methods utilised by the clinicians.

*Like there’s been cards to show how to do breathing and there’s been worksheets and sometimes she gave me a booklet to colour in. Like all different activities to do.*

(YP05)

*So when I’m straight talking, I don’t really like it and I feel less comfortable, but if I’m drawing it gets my mind off that and I just let everything out.*

(YP08)

Parents also appreciated that the service could be individualised for the needs of each young person:

*I liked that she’s been a bit creative about things like, you know, [YP] is a drawer, so she sometimes gets [YP] to draw how she’s feeling and things like that. She’s sort of just taken the time to get to know her and find out what will work to get her to talk; not just a blanket sort of thing, it’s actually specific to her needs.*

(FM03)

### 3.9. Confidentiality

The young people appreciated that they had input into when their parents or other professionals were involved and what was said to them:

*I really like the fact that it is just us [young person and clinician]. We decide when we want to talk. And… we make all the calls on that stuff [who else to involve].*

(YP06)

The parents interviewed all felt that the service had struck a good balance between communicating with them and maintaining a confidential space for the young person:

*And if there’s anything that comes up that needs addressing, I like how that gets fed back to us, but I also like that the girls are given their own privacy to have their sessions and that I’m not part of them at all.*

(FM06)

They felt that the clinician remained available to speak to them throughout the process and that they had an ally in their concerns and hopes for their child. They had confidence that the service clinician would contact them if needed:

*I knew that [clinician] had the girls’ best interests at heart and if I needed to know anything she would let me know.*

(FM08)

### 3.10. Networks

As well as having individual contact with the service, young people described clinicians offering them resources and contacts for other support services, and, in some cases, facilitating a connection with other clinicians:

*My anxiety was making me physically ill… She [clinician] asked if she could refer me to a dietician… She came with me… and it was really good. I learned a lot from that.*

(YP06)

*I’ve liked the fact that she [clinician] helped me get a psychologist to help me with my behavioural and attention problems and I might have a full diagnosis.*

(YP07)

Family members also identified benefits from the networks generated by the service.

*[Clinician] was very instrumental in suggesting the doctors and the psychologist we ended up seeing in [town]… She attended those visits with [YP] when I was unable to get there… All that support, just being able to attend the appointment, and for me to be able to see [YP] through [clinician] eyes, what she saw, all with his permission to share information. I know that everything wasn’t shared because it didn’t need to be, but it was really good to have someone there helping him open up.*

(FM05)

## 4. Experiences of Change

The aim of a mental health support service is to improve the mental state of individuals. This study asked if anything changed (positive or negative) for the young person or family as a result of using the service. Young people described their perceptions of change, while parents described the change for the young person as well as for themselves or their family.

### 4.1. Young People’s Perceptions of Change

The young people reported improvements in their mental health because of contact with the service:

*Like with my strategies and stuff, it’s helped me with my depression and anxiety, and it’s made my life better … So before I went to [clinician], I had a lot of mental problems and I couldn’t really do anything and I didn’t know how to help myself. And then when I came to [the service], [clinician] helped me with a lot of my depression stuff and the strategies. And whenever I have those moods, I remember what she talked about and it helps.*

(YP08)

*Just I’m a whole different kid pretty much. … I feel more open to talking about other things, because I’m so used to talking about them now…. I’m not moping around [at home] all the time … I’m doing some stuff instead of just lying in bed*

(YP05)

Specifically, the young people reported positive changes in their confidence and level of social interaction:

*I’m a lot happier, just like in general. A lot more confident in myself. It’s made it like for me I’m I can talk to people a lot easier now. I’ve found like I’ve been a lot more positive about life and it’s made my life a lot easier…. It’s just put a big impact on my life. Like, it’s just amazing.*

(YP02)

Also improved were peer relationships:

*I wasn’t really involved [in school]–I had only a couple friends at school ‘cause I don’t really like… wanting to socialise with people, just ‘cause of my anxiety, and past things that have happened with people. So [clinician] has helped me branch out. I’ve got more friends at school now, and I do more things with school, and just get more involved which I didn’t do before.*

(YP11)

Family relationships were also improved:

*I talk to my family about a lot more things. [Clinician] helped me through ways to talk to my family. That was really good.*

(YP03)

### 4.2. Family Members Perceptions of Change

Each of the parents interviewed described positive changes for their young people which were attributed to their contact with the service. Paralleling the reports of the young people, parents described improvements in mood, anxiety management, communication, and family relationships:

*She [YP] has changed probably in the last eight months… She has been more open. She has engaged in the family a lot more. She’s not fighting with her younger siblings… she’s starting to get a better understanding of how to control her emotions. It’s opened up channels of communication with me and [YP] that I didn’t know how to do… [clinician] has done a wonderful job with teaching [YP] all these tools–it’s really helping.*

(FM02)

*Definitely a big change in communication, huge change in her moods even, she’s just a lot happier, she’s a lot more relaxed, and she does seem to have the strategies now when she’s not feeling so good… She’s a different kid at the moment. We’ve been talking over the last few weeks about how nice it is to be around her again, having her being the normal her again, the happy her.*

(FM03)

Improvements in mental health for the young people translated into functional improvement and allowed for the attainment of life goals:

*We are miles ahead of where we were. [YP] is now employed. He has his license-he turned 18 last year and was unable to have the confidence to do the tests and the driving. He’s a year behind but he’s now achieved his license, he’s an independent driver and he has a part-time casual job. That job was actually mentioned to him by [clinician]. He has progressed in the last few months probably to a point that we never thought we’d ever get to. I know for a fact we wouldn’t be there without [the service] being involved…. giving [YP] his confidence back, bringing that child back to who he was after a very bad incident.*

(FM05)

## 5. Discussion

This study explored the experiences of young people and family members who had accessed a rural, mobile mental health support service. The results indicate that both young people and their family members had a positive experience with the service because of its ease of access and flexibility and its approachable and responsive clinicians. Further, both groups of participants could describe positive impacts on the mental health and wellbeing of the young people, which they attributed to the support service. A variety of specific strategies and techniques used in the sessions were described, and examples of changes in mental state were provided. Importantly, even though the support service was not at every site every day, the perception of ready availability came through strongly in the accounts of the participants. Clearly, participants valued the flexibility of the service in using multiple methods of communication, including texts and emails, and young people appreciated the autonomy to use the service how they wished.

The key references participants made to the rural context were around finances and distance. The support service was free, and several participants noted that any other service would have cost money to attend, which would preclude accessing them. Many health services in Australia are free or subsidised by the government health system. However, specialist mental health care is frequently provided by private practitioners who charge a fee. Incomes in rural Australia are lower on average when compared to urban areas, and this lower income impacts all the social determinants of health [5]. The need to travel long distances to access care is also related to the cost of care relative to income. Participants noted that the school location of the support service was convenient for the young people and for their families, as they did not have to cover fuel costs to travel, either from home to the service or to other towns, or take time off work to access the service.

The use of mental health services in rural areas has been described as limited by the knowledge people have about their conditions, particularly adolescents [1]. This was not supported in this study. While young people and their parents may not have had diagnostic terminology for their experiences, instead referring generally to emotional or behavioural problems, there was an awareness of needing professional help. However, there was a lack of knowledge by young people about the service system and in what ways support could be accessed. The location of the service was an important factor here. School was a place the young people regularly attended, did not require parental involvement to access, and could be facilitated by teachers or school counsellors. The location was possibly a barrier to accessing support for young people who were not attending school, although meetings in locations outside the school were noted. This may explain the low take-up of the service by the upper end of its target age range, 12–25 years.

Once engaged with the service, the study participants supported the findings of previous studies regarding enablers and barriers to support [23]. Experiences of kindness, responsiveness, multiple means of communication, choice, and control over when, where, and how often to attend the service were all noted as important factors in the satisfaction of the study participants. Other factors in the way the service was organised, such as easy referral and assessment processes, trust in confidentiality, and connections with the clinicians, addressed the barriers to support that were identified in the literature [22,24]. These factors should be considered in the user-led design of new services [27] to prevent the barriers being continually re-created. While the study was conducted in western NSW, Australia, it reflects the findings of earlier, diverse research on the access and use of mental health services e.g., [22,23,24,25,26]. The findings have implications for changing the way that these services are organized and operated. Healthcare policies and services could support a user-designed model that incorporates the access and use enablers and removes the barriers to rural mental health support. Further, many of the elements that young people valued, such as kindness, responsiveness, an availability of multiple means of communication, choice, and control, are just as likely to apply to mental health supports for other age groups.

The switch to virtual care because of restrictions on travel and contact due to COVID-19 appeared to cause little disruption to the service. The young people expressed varying personal preferences for on-line or face-to-face support. This is consistent with the results of studies examining the provision of virtual care [16]. The experiences and expertise of clinicians with virtual care continue to grow [13], although the reports in this study of access to care virtually depict a seamless transition from the perspectives of the young people and their families. Similarly, the experiences of young people with virtual care are still limited [14]. While the experiences of young people in this study suggest that virtual service provision may be a useful way of increasing access to mental health services in rural areas, more research is needed to investigate the outcomes and experiences of virtual care when compared to in-person care.

### Strengths and Limitations

A strength of this study was that it provided the opportunity to hear directly from rural young people and their carers about their experiences with receiving mental health care. However, given that participation was voluntary, the young people self-selected to participate in the study. All the young people interviewed were current clients of the service. Thus, the voices of young people who knew about the service but did not access it, and those who had discontinued service contact, were not heard. Furthermore, the self-selected sample significantly under-represented First Nations service users. Nevertheless, the perceptions of the participants included were highly consistent across both cohorts of young people and their parents. Further research is required to assess the effectiveness and outcomes of the service and to address the limitations of the current study.

## 6. Conclusions

According to young people and their families, the mobile-outreach service model is effectively meeting the challenge of providing mental health support to dispersed and under-serviced young people in rural and remote areas. The key enablers facilitating access were the flexibility of the service, the variety of service-delivery modes and therapeutic methods offered by clinicians, the ease of access facilitated by the location in schools, and the autonomy of the young people to choose how they utilised the service. In the context of increasing workforce supply challenges, these appear to be key factors in providing appropriate and effective mental health support to young people across rural areas. Healthcare policies and services could support a user-led model design that incorporates access and use enablers and removes the barriers to rural mental health support.

## Data Availability

The data presented in this study are available on request from author J.A. with the permission of author A.T. The data are fully reported in English and are not publicly available due to privacy/ethical restrictions.
